# The Electrical Properties of Hybrid Composites Based on Multiwall Carbon Nanotubes with Graphite Nanoplatelets

**DOI:** 10.1186/s11671-017-2168-8

**Published:** 2017-06-13

**Authors:** Yulia Perets, Lyudmila Aleksandrovych, Mykola Melnychenko, Oleksandra Lazarenko, Lyudmila Vovchenko, Lyudmila Matzui

**Affiliations:** Department of Physics, Taras Shevchenko National University of Kyiv, Volodymyrska Str., 64/13, Kyiv, 01601 Ukraine

**Keywords:** Polymer composite, Hybrid filler, Graphite nanoplatelets, Multiwall carbon nanotubes, Percolation threshold, Electrical conductivity, Thermal conductivity, 72.80.Tm, 64.60.ah, 82.35.Np

## Abstract

In the present work, we have investigated the concentration dependences of electrical conductivity of monopolymer composites with graphite nanoplatelets or multiwall carbon nanotubes and hybrid composites with both multiwall carbon nanotubes and graphite nanoplatelets. The latter filler was added to given systems in content of 0.24 vol%. The content of multiwall carbon nanotubes is varied from 0.03 to 4 vol%. Before incorporation into the epoxy resin, the graphite nanoplatelets were subjected to ultraviolet ozone treatment for 20 min. It was found that the addition of nanocarbon to the low-viscosity suspension (polymer, acetone, hardener) results in formation of two percolation transitions. The percolation transition of the composites based on carbon nanotubes is the lowest (0.13 vol%).

It was determined that the combination of two electroconductive fillers in the low-viscosity polymer results in a synergistic effect above the percolation threshold, which is revealed in increase of the conductivity up to 20 times. The calculation of the number of conductive chains in the composite and contact electric resistance in the framework of the model of effective electrical resistivity allowed us to explain the nature of synergistic effect. Reduction of the electric contact resistance in hybrid composites may be related to a thinner polymer layer between the filler particles and the growing number of the particles which take part in the electroconductive circuit.

## Background

Using several fillers simultaneously (mostly mixtures) is the trend of recent years since it can significantly improve the properties of produced composite materials (CMs), such as electrical and thermal conductivity, elastic properties—strength, Young’s modulus, glass transition temperature, and mechanical losses as compared with CM with a single filler. The addition of a multi-component filler to a polymer matrix promotes interaction between these fillers. Thus, improved conductivity as a result of synergistic effect was observed in CMs based on polyethylene with graphite particles and carbon fibers (CFs) [1] as well as in CMs containing carbon black and CFs [2, 3]. The mechanism of the conductivity enhancement consists in double percolation and represents the function of CFs in enhancement of the connectivity of conductive paths. The coexistence of two conductive nets formed by carbon black particles and carbon fibers reinforcing each other, leads to a significant improvement in the electrical characteristics of the CM, since fibrous filler interacts with spherical particles of carbon black which stimulates the formation of conductive network in a polymer matrix.

Classical percolation with one sharp transition from a non-conductive to a conductive state is commonly expected for composites filled with highly conductive particles. So far, a lot of different models and equations were proposed for a description of the conductivity behavior [4, 5].

However, in many experimental observations, the percolation in composites is more complicated. The presence of two-step (double percolation), several-step (multiple percolations), and even fuzzy (smeared) type of percolation transitions has been reported [6–12]. The character of percolation threshold is determined by the distribution of particles, its types, and types of the electrical contacts, geometrical effects, and selective distribution of conductive particles in multi-component media (e.g., in polymer blends). The existence of static and kinetic network formation processes, as well as the core-shell structure of particles, may be responsible for the multiple percolation thresholds.

Synergistic effect can appear in improvement of the electrical or thermal properties of the CMs, even when one of the fillers is not highly electrically or thermally conductive. In [13], Kim et al. investigated CMs based on polyether ether ketone (PEEK) with hybrid SiC and CF fillers. Significant improvement in the thermal conductivity of the CM was observed, which is the result of the formation of effective thermal paths in the CM.

There are recent papers which present the results of research of the composites with nanoscale fillers [14, 15] and its mixtures. Thus, it was shown in [16] that the addition of carbon nanotubes (CNT) in the CM with carbon black increases conductivity of CM. In addition, carbon black particles also increase the viscosity and crack resistance of nanocomposites, hence confirming a synergistic effect of carbon black as a multifunctional filler. In [17, 18], Zhao et al. investigated composites with carbon nanotubes and graphite nanoplatelets (GNPs). Low percolation transition was observed due to improved interaction between different carbon fillers as a result of a modified process of the samples manufacture. Not individual particles of carbon fillers are added to the polymer, and graphite nanoplates on which carbon nanotubes are grown and aligned. These structures are considered as one whole hybrid particle, it has a complex morphology.

We had investigated the hybrid polymer composite materials consisted of conductive and dielectric components [19, 20]. Results showed that the dielectric filler exfoliates graphite nanoplatelets and untangles carbon nanotubes in solution of resin in acetone solvent during the manufacturing of composites. This led to improved electrical and thermal properties of the samples.

Hybrid polymer composites are very topical now. But do all combinations of various fillers and various polymers lead to positive results? Of course not! Firstly, few researches have been conducted in this area; secondly, theoretical simulations of various hybrid systems and their properties show excellent results, but they are not always confirmed experimentally [21].

The novelty of this work is that for achievement of synergistic effect, two conductive fillers with the unique geometric shape and different aspect ratios as well as the different dispersion characteristics are used.

## Methods

### Materials

**Fig. 1:**
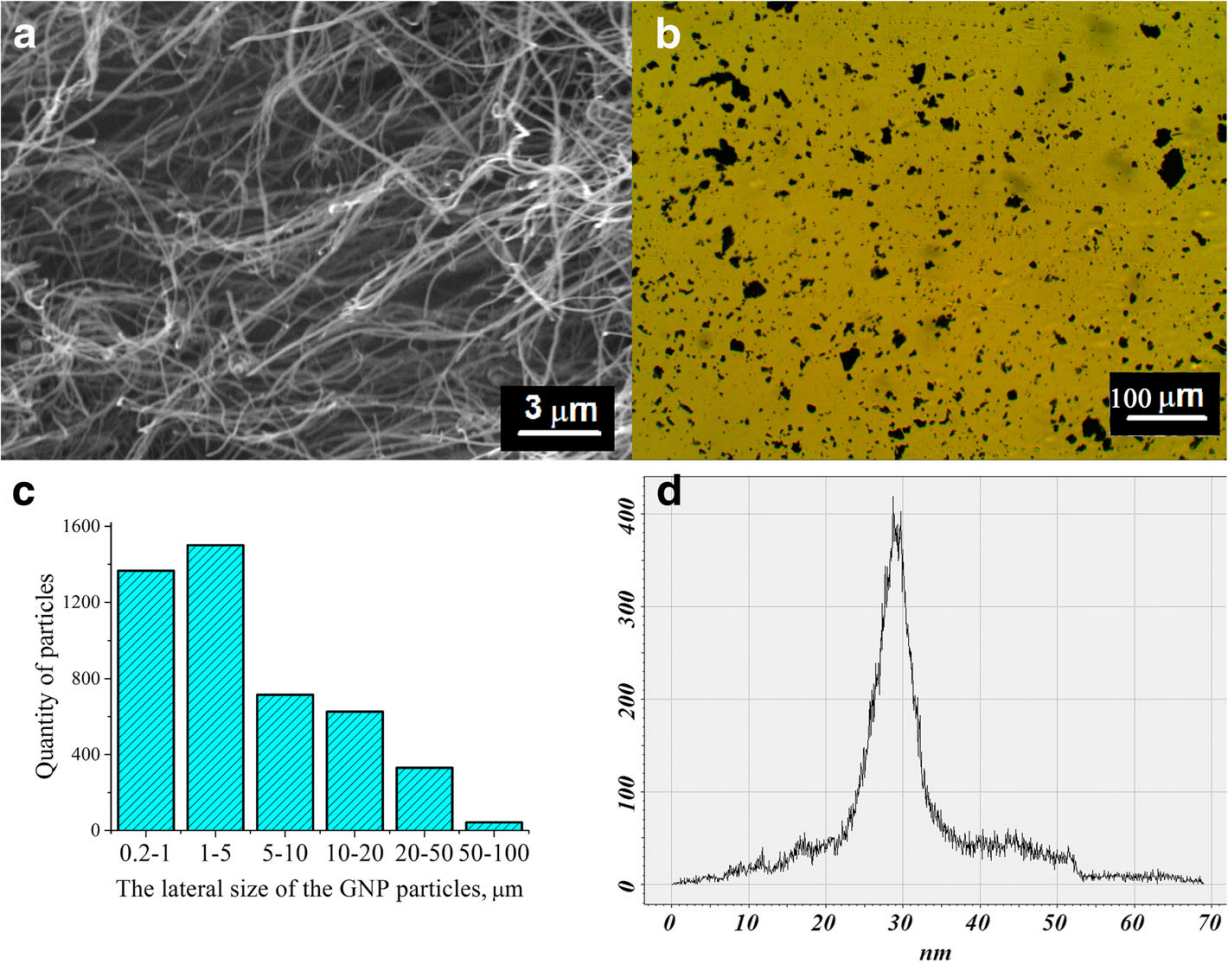
SEM images for the MWCNT (**a**). Optical image for the GNP (**b**). The distributions of particles (diagram) on the lateral size of TEG after 30 h ultrasonic dispersion in water (GNPs) (**c**). Histograms of thickness distribution of GNPs obtained in dispersive mediums—in water (**d**)

Figure 1a presents SEM image of used multiwall carbon nanotubes (MWCNTs) with purity ≥90% (Cheap Tubes Ins.). Optical microscopy image of GNPs that was used as the second filler is shown in Fig. 1b.

Thermally expanded graphite (TEG) is a product of natural disperse graphite (*d* = 50–300 μm, *h* = 5–30 μm) intercalation with H_2_SO_4_ and subsequent heat treatment in a furnace with ascending flow according to the method developed and it was reported in previously published paper [22]. The peculiarity of the TEG ultrasonication process in the water medium is that the TEG particles do not sink but float on the surface. For this reason, TEG exfoliation into GNPs is complicated. After 30 h of TEG ultrasonication in the water medium, the most part of the GNPs are of 0.2–5 μm in diameter; however, big particles of 10–100 μm in diameter occur as well (Fig. 1c).

On the basis of 3D-converted AFM images of GNPs obtained in different dispersive mediums, we carried out a comparative analysis of GNP thicknesses. The histograms of thickness distribution are presented in Fig. 1d. Based on AFM results, the variation of thickness distribution for GNPs (obtained in water medium) was 5–55 nm with the maximum of distribution at 28 nm. The estimation of lateral dimensions allowed the calculation of aspect ratio of GNPs which is of ~40–900 for GNPs. Therefore, a conclusion can be made that the GNPs obtained in water dispersive medium possess the wide distribution of thicknesses and lateral dimensions. This is certainly favorable for higher electrical conductivity of the composite with the filler of this kind.

Structural and morphological characteristics of investigated fillers are presented in Table 1. As can be seen in Table 1 and Fig. 1, the shape of a nanocarbon filler considerably differs. Thus, GNPs can be considered as disks and MWCNTs as cylinders.

**Table 1:** Structural and morphological characteristics of fillers

	GNPs	MWCNTs
Shape	Disks	Cylinders
Length, *l*	–	10 μm
Diameter, *D*(*d*)	0.2–50 μm	10–30 nm
Thickness (*h*)	5–55 nm	–
Aspect ratio, *A*	40–900	330–1000

### Preparation of Composites

This paper presents the results of the investigation of changes of electrical resistivity and thermal conductivity of hybrid polymer composites with multiwall carbon nanotubes (MWCNTs) upon the addition of constant amount of the second electrically conductive disk-shaped filler—graphite nanoplatelets.

For the study of electrical properties of carbon-epoxy resin polymer composites, two systems have been prepared:

- ➢Two-component system, where GNPs or multi-walled MWCNTs were used as fillers—*mono composite materials* (MCMs)
- ➢Three-component system, where electrically conductive filler GNPs was used as the second filler for CMs with MWCNTs—*hybrid composite materials* (HCMs)

#### Mono Composite Materials

During our work, we synthesized and investigated the composite systems based on epoxy Larit 285 (Lange Ritter GmbH, Germany). This resin has the following characteristics: epoxy equivalent = 165–170, epoxy number—0.59 ÷ 0.65.

In order to prepare nanocarbon/epoxy MCMs, nanocarbon fillers were incorporated into epoxy resin Larit 285 (viscosity of 600–900 mPa s) with H285 (viscosity—50 ÷ 100 mPa s, amine number—480 ÷ 550 mgКOH/g) as a hardening agent. The content of the nanocarbon filler in MCMs varied from 0.03 to 4 vol%.

The GNP powder was subjected to UV/ozone treatment (for mono and hybrid CMs). UV/ozone treatment was performed by using of lamp DRT-1000. The initial GNP powders were subjected to UV/ozone treatment for 20 min [22, 23].

Three grams of the epoxy Larit 285 were placed in the test tube for further dissolution in the acetone solvent. Powder-like nanocarbon had weighed out for selected concentration and added to epoxy–acetone solution. The nanocarbon filler (GNPs or MWCNTs) was mechanically mixed with epoxy resin and acetone. A mixture of these components was stirred during 30 min (for GNPs) or 60 min (for MWCNT) in an ultrasonic bath for more uniform distribution of the filler in the polymer, then the curing agent H285 was added, and a mixture was poured into molds and cured at room temperature for 48–72 h to complete the polymerization.

#### Hybrid Composite Materials

MWCNTs were used for the preparation of HCMs, as a main electroconductive filler with varying concentrations from 0.03 to 4 vol%. To study the synergetic properties of the additional dispersed electric filler, GNPs were added to the given systems in a content of 0.24 vol%.

MWCNTs were mixed with epoxy resin and acetone. A mixture of these components was stirred during 60 min in an ultrasonic bath for more uniform distribution of the filler in the polymer. Then, GNP powder was added and thoroughly mixed mechanically, and all were stirred during 30 min in an ultrasonic bath. Then, the curing agent H285 was added, and a mixture was poured into molds and cured at room temperature for 48–72 h to complete the polymerization.

For the measurements of electrical conductivity, the samples with a shape of rectangular parallelepiped with a size of 3.5×3.5×10 mm^3^ were prepared. The measuring conductivity range was from 10^−12^ to 10 S/m.

### Methods of Testing

Ultrasonic dispersing of TEG powder was carried out in ultrasonic bath “BAKU” BK-9050, US frequency—40 kHz, with a maximum electrical power output of 30 and 50 W. The lateral dimensions of the prepared GNPs were investigated by using an optical microscope MIKMED-1 with the attached digital camera ETREK DCM-510 and probe NanoLaboratory INTEGRA. To estimate the average thickness and diameter of the GNPs, their optical and atomic-force microscope (AFM) images were converted into 3D images by program Nova, which created the histograms of GNP density distribution.

UV/ozone treatment was performed by using DRT-1000 (ultraviolet lamp) equipped with electric-discharge arc lamp of high pressure inflated with mercury and argon compound that could release ultraviolet radiation of 50 W at 240–320 nm wavelength. The distance between the UV lamp and the sample was fixed at 11 cm.

The electric resistance of the samples was measured by two-probe (*R* = 10^4^–10^9^ Ω) and four-probe (*R* ≤ 10^4^ Ω) method or by teraohmmeter E6-13 (*R* = 10^9^–10^13^ Ω). An automated installation was used for the investigation of the electric resistance in the temperature range of 6–300 K. The main components of the automated installation were a rod for a sample, a power switching current direction and a stable source of voltage, an analog–digital converter ADC 16-32F (SDI), a personal computer, and the interface cables. The temperature was measured by a copper–constantan thermocouple located near the sample. The measurement range of electric resistance (0.01–10^14^ Ω) was divided into several regions: 0.01–2.5 Ω, where error does not exceed 0.5%; 2.5–10^7^ Ω (error was <1%); *R* = 10^8^ Ω (<5%); *R* = 10^9^ Ω (<10%); *R* = 10^10^–10^13^ Ω (<20%). When measuring the electric resistance of CMs, three samples for each concentration were tested.

## Results and Discussion

### Electrical Conductivity of the Polymer Composites with Mono and Hybrid Fillers

**Fig. 2:**
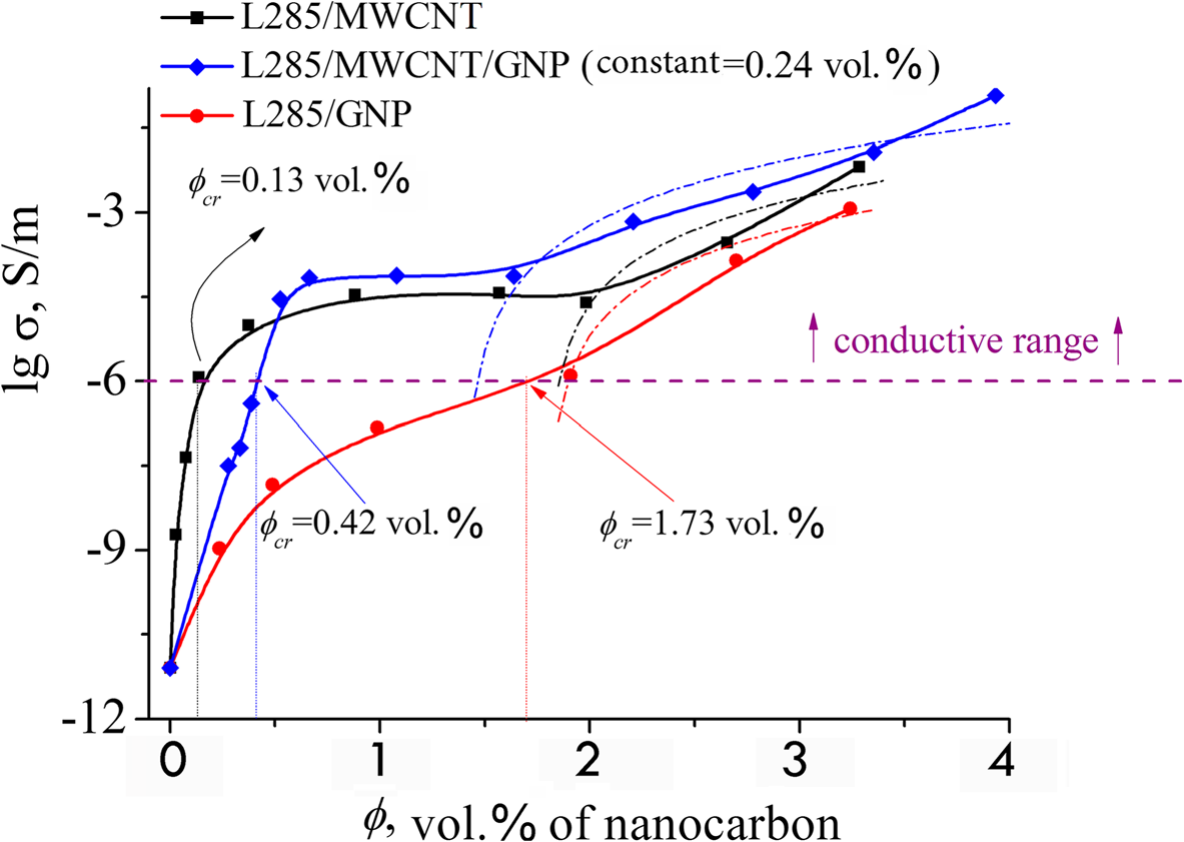
Electrical conductivity of composites as a function of nanocarbon content. *Solid line* the experimental curves; *dotted line* the calculated curves according to Eq. (1)

The percolation threshold *ϕ*_cr_ was investigated using volume conductivity measurements. The dependence of electrical volume conductivity of prepared composites on the filler content is shown in Fig. 2. The values for the lowest concentrations correspond to the pure epoxy resin conductivity of 7.9 × 10^−12^°S/m.

As it can be seen from Fig. 2, the concentration dependences of conductivity for both MCMs and HCMs have two percolation transitions. The lowest (at 0.13 vol%.) was found for samples with MWCNTs. Starting at 0.137 vol% of MWCNTs, the values of conductivity are higher than 10^−6^ S/m representing the conductive range. For samples based on GNPs, the highest critical concentration *ϕ*_cr_ equals 1.7 vol% is observed, and there is only one percolation transition above the conductivity value of 10^−6^ S/m. For samples with the mixed fillers with constant concentration of GNPs (*ϕ* = 0.24 vol%.), the critical concentration of MWCNT/GNP was found to be equal *ϕ*_cr_ = 0.42 vol% and lain between the values of the composites with pure fillers.

Similar behavior of *σ*(*ϕ*), namely, the presence of two percolation transitions on the concentration curve was observed for a number of composites [24–27].

Josef Z. Kovacs and others [24] consider that such percolation thresholds induced by kinetic processes and therefore cannot be determined using the common percolation scaling law from statistical percolation theory.

Also, double percolation transition has been obtained by Mamunya and others in [25]. The authors also used the polymer–carbon nanotubes composites, but they combined two polymers (copolyamide and polypropylene) as a polymer matrix, which they mixed with the filler at high temperatures (125, 167 °C) and pressed at 180 °C.

We suppose that the existence of two percolation thresholds in our work is a characteristic feature of composite materials which have a low viscosity at the stage of sample manufacture (Fig. 2 and Table 2).

**Table 2:** Percolation characteristics of nanocarbon–polymer CMs with GNP, MWCNT, and hybrid filler–MWCNT/GNP

Filler	Polymer	*ɸ*_cr_, vol%	*ɸ*_cr_, vol% (static)	*t*
GNP	L285	-	1.80	2.52
MWCNT	L285	0.13	1.80	2.42
MWCNT + GNP (*ϕ* = 0.24 vol%)	L285	0.42	1.40	2.84

We believe that the first percolation transition can be considered as a quasi-dynamic percolation transition by analogy with the dynamic percolation transition observed in polymer–carbon composites, where the percolation transition is formed under the action of external forces (electric or magnetic field, pressure, etc.) [24, 27].

In the low-concentration region, after the addition of hardener, the liquid polymer with nanocarbon has a low viscosity. The particles of the carbon filler in the polymer matrix can be represented as sufficiently large agglomerates of nanoparticles (even despite long-duration ultrasonic dispersion) and separate nanoparticles (nanotubes or GNPs) with sufficiently high mobility in a low-viscosity polymer matrix. Under the action of van der Waals or electrostatic forces, these separate nanoparticles (nanotubes) can move connecting with each other as well as with large agglomerates of nanoparticles. Thus, due to this displacement, until the polymer matrix hardens, these separately mobile nanoparticles can form conductive chains that provide the conductivity of the whole sample.

The formation of the “shelf” or plateau after the quasi-dynamic percolation threshold in concentration dependence of electrical conductivity for CMs with MWCNTs and hybrid filler (where CNT concentration dominates) depends on several factors. It is primarily related to the increase of the filler concentration, which leads to an enhancement of the viscosity of uncured sample and restricted movement of the separate particles to form new conductive chains. Secondarily, the number of separate mobile particles also increases. Besides, simultaneous increase of the viscosity and the number of capable to efficient movement individual nanoparticles decelerates the process of formation of the conductive chains, and, consequently, the conductivity growth with increasing filler concentration. It is seen from Fig. 2 that in order to achieve the second critical concentration, it is required to add a significant amount of filler to CM. Then, single particles start the interaction with each other as well as agglomerates of particles and agglomerates of particles with a single CNT.

**Fig. 3:**
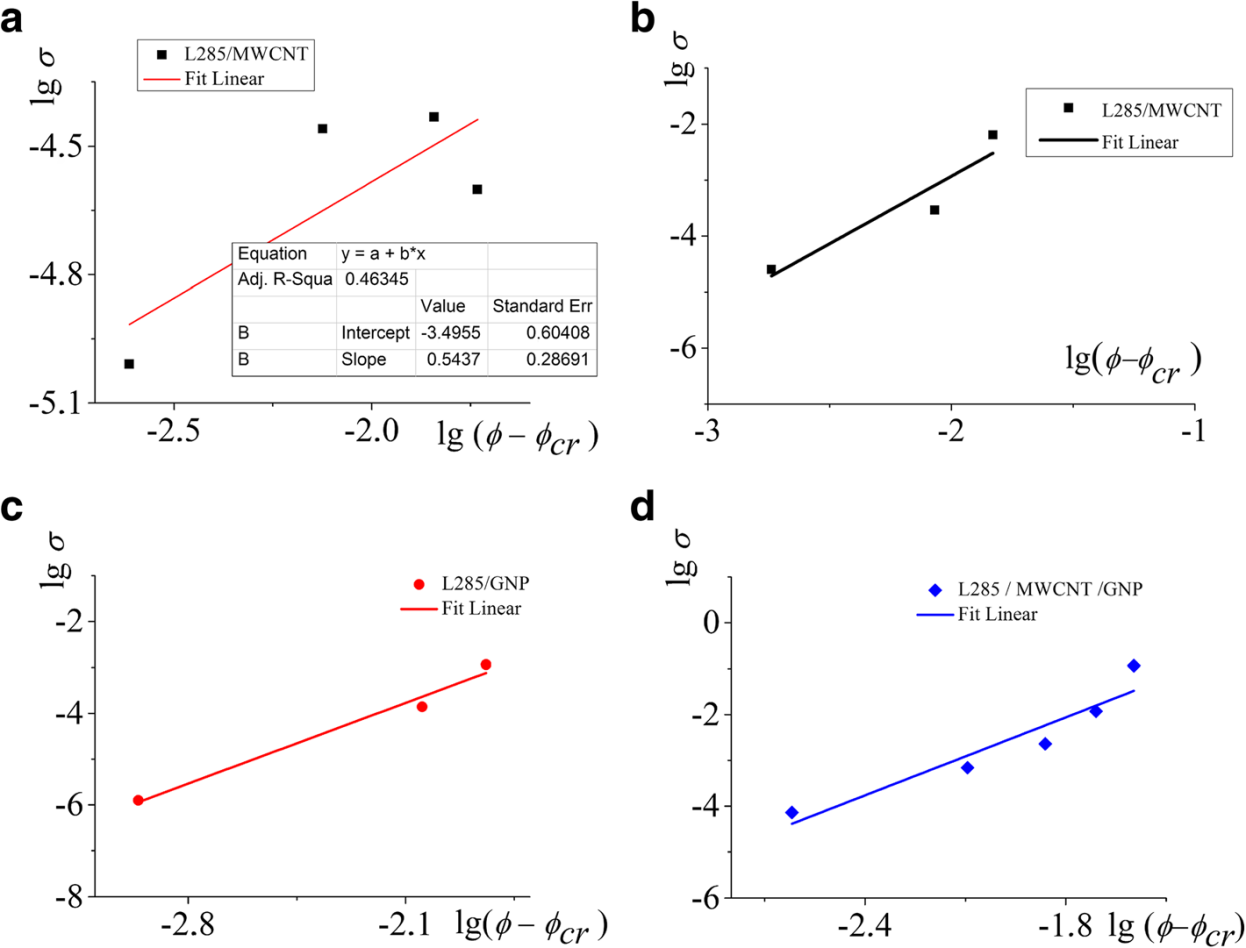
**a**–**d** Scaling dependence (lg*σ* as a function of lg*(ϕ − ϕ*_cr_)) for determining the parameters of Eq. (1). **a** The quasi-dynamic percolation transition, **b**, **c**, **d** The statistical theory of percolation

Quasi-dynamic percolation transition cannot be described in the framework of the classical percolation theory (Fig. 3a). The second percolation transition is defined and described by the statistical theory of percolation (Fig. 3b–d) [28, 29]:

1 $$ \sigma \left(\phi \right)\sim B{\left(\phi -{\phi}_{\mathrm{cr}}\right)}^t $$

where *ϕ*_cr_ is the critical concentration (percolation threshold), *t* is the critical index, and *B* is the proportionality constant in the classical percolation model. The constants *B*, *ϕ*_cr_, and *t* were fitted using the method of mean squared error minimization (Fig. 3, Table 2). The fitted values of *ϕ*_cr_ are included in Fig. 2.

**Fig. 4:**
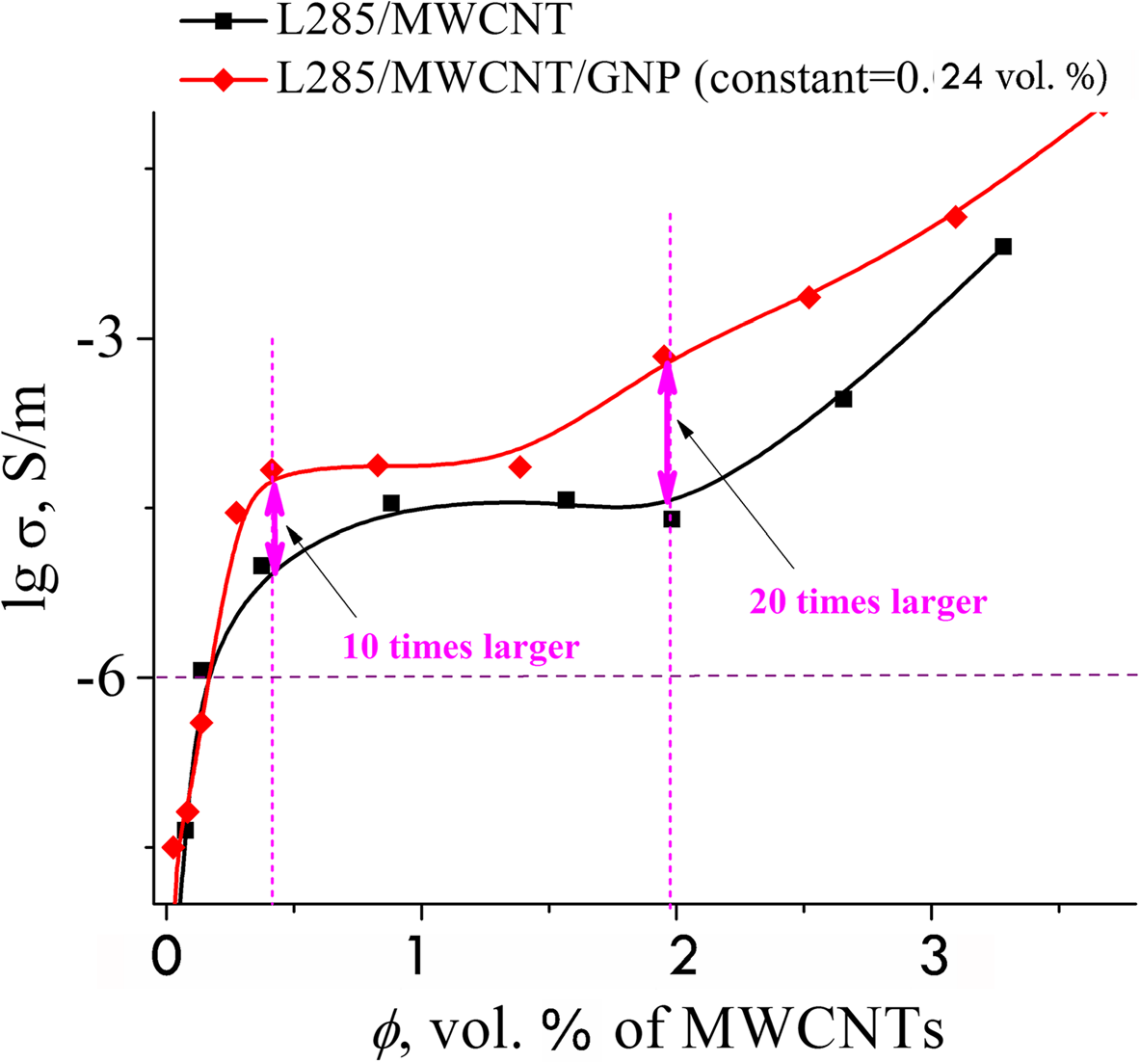
Electrical conductivity of composites as a function of MWCNTs content

In Fig. 4, we present the electrical conductivity versus the concentration for MCM–L285/MWCNT and HCM–L285/MWCNT/GNP. As one can see in the picture, the percolation transition is the same for both composites. A synergistic effect is observed as enhancement of the electrical conductivity of HCM above the critical concentration (Fig. 4). The greatest synergistic effect was observed in CM with the combination of two electrically conductive fillers—carbon nanotubes and graphite nanoplatelets—and at concentration 2 vol%, the electrical conductivity is 20 times higher and at 4 vol%, 10 times higher as compared with MCM.

**Fig. 5:**
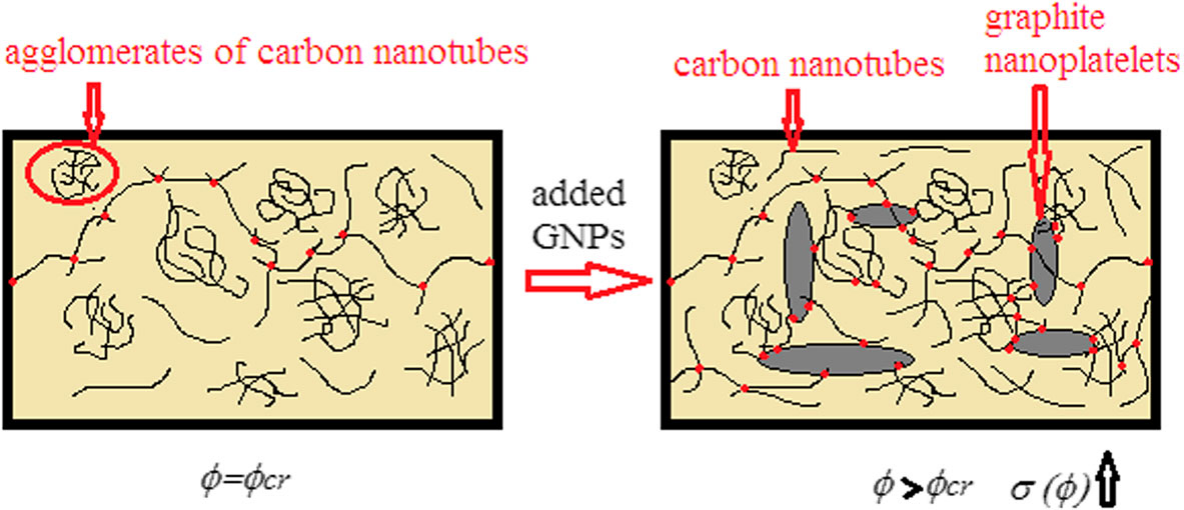
Principles of conductive pathway formation in hybrid CM–L285/MWCNT/GNP

To understand the mechanism of the formation of conductive chains in the hybrid CM where synergistic effect is observed, we illustrate the possible scheme in Fig. 5. To untangle the bundles of CNTs, they are subjected to ultrasonic dispersing. As a result, not all CNTs are unraveled, besides they break up reducing their aspect ratio; thus, the number of CNTs required for the formation of the conductive chains increases.

In order to achieve a synergistic effect, we conceived to add a small amount of GNP particles into a composite with CNT for the connection of not fully unraveled agglomerates of CNTs. Due to the liquid medium (namely, a low-viscosity resin) and a quasi-dynamic percolation, we failed to reach percolation threshold lower than that in mono CM with CNTs. We can explain it only by the fact that in the case of ultra-low percolation transition (0.13 vol%), the continuous conductive chains are formed not in large numbers (it is seen from Fig. 4, since the conductivity values are low at the first percolation transition) mainly from separate CNT particles, which are not bundled and still do not have contact with the GNP particles. After the percolation threshold, a significant increase of conductivity of the hybrid CM is observed due to the additional chains of agglomerates of CNTs and GNPs (Fig. 5).

Morphological features of the nanocarbon conductive component of the filler are critically important during the formation of conductive chains. Total amount of chains (hence, contact resistance between the particles and percolation characteristics) in the CM is affected mainly by the shape of filler (skeleton form of CNT and chain-like form of GNP). In order to investigate the number of chains and the contact resistance between the particles in the CM, we utilized a model of the effective electric resistance.

In terms of the proposed model, the electric resistance of the chain consisted of a nanocarbon particles which are as follows [30]:

2 $$ {R}_{\mathrm{CM}\_\mathrm{G}\mathrm{N}\mathrm{P}\left(\mathrm{MWCNT}\right)}=\frac{N_{\mathrm{GNP}\left(\mathrm{MWCNT}\right)\_\mathrm{in}\_\mathrm{chain}}}{N_{\mathrm{chain}\_\mathrm{in}\_\mathrm{C}\mathrm{M}}^{*}}\cdot \left({r}_{\mathrm{GNP}\left(\mathrm{MWCNT}\right)}+{R}_K\right) $$

where ${N}_{\mathrm{GNP}\left(\mathrm{MWCNT}\right)\_\mathrm{in}\_\mathrm{chain}}=\frac{b\left(1\kern0.5em \mathrm{cm}\right)\cdot \gamma}{l}=\frac{\gamma}{l}$ is the amount of the nanocarbon particles in one chain, *b* is the length of the sample (1 cm), *γ* is the constant factor with value from 1 to 2, *l* is the length of the nanocarbon particle (GNP or CNT), *r*_GNP(MWCNT)_ is the electric resistance of the filler particle (for the disk-like particles—${r}_{GNP(disk)}={\rho}_{GNP}\cdot \frac{d}{d\cdot h}=\frac{\rho_{GNP}}{\mathrm{h}}$, for the cylindrical one’s—${r}_{\mathrm{MWCNT}\left(\mathrm{cylinder}\right)}={\rho}_{\mathrm{MWCNT}}\cdot \frac{4 l}{\pi {d}^2}$), *h* is the thickness of the nanocarbon particle, *d* is the diameter, and *R*_*к*_ is the electric resistance of the single contact between particles of the filler (CNT or GNP).

Summing up, the electric resistance of the polymer/nanocarbon CM can be evaluated as [30]:

3 $$ {R}_{\mathrm{CM}\_\mathrm{G}\mathrm{N}\mathrm{P}\left(\mathrm{MWCNT}\right)}=\frac{N_{\mathrm{GNP}\left(\mathrm{MWCNT}\right)\_\mathrm{in}\_\mathrm{chain}}}{N_{\mathrm{chain}\_\mathrm{in}\_\mathrm{C}\mathrm{M}}^{*}}\cdot \left({r}_{\mathrm{GNP}\left(\mathrm{MWCNT}\right)}+{R}_K\right)=\frac{\gamma^2\pi \cdot z}{4 F}{\left(\frac{F-{\phi}_{\mathrm{cr}}}{\phi -{\phi}_{\mathrm{cr}}}\right)}^t\cdot \left({r}_{\mathrm{GNP}\left(\mathrm{MWCNT}\right)}+{R}_K\right) $$

where *N* ^*^_chain_in_CM_ is the number of a parallel-connected nanocarbon chains. This number is proportional to the total amount of particles, participating in the electroconducting. *F* is the packing factor (*F* = 0.05 for GNP and *F* = 0.06 for CNT), *z* = *h* is for GNP, and $z=\frac{d^2}{l}$ is for CNT. This model takes into account not only the critical concentration *ϕ*_cr_, packing factor *F*, and electrical resistivity of the filler *r*_GNP(MWCNT)_ but also the morphology of the particles (aspect ratio).

**Fig. 6:**
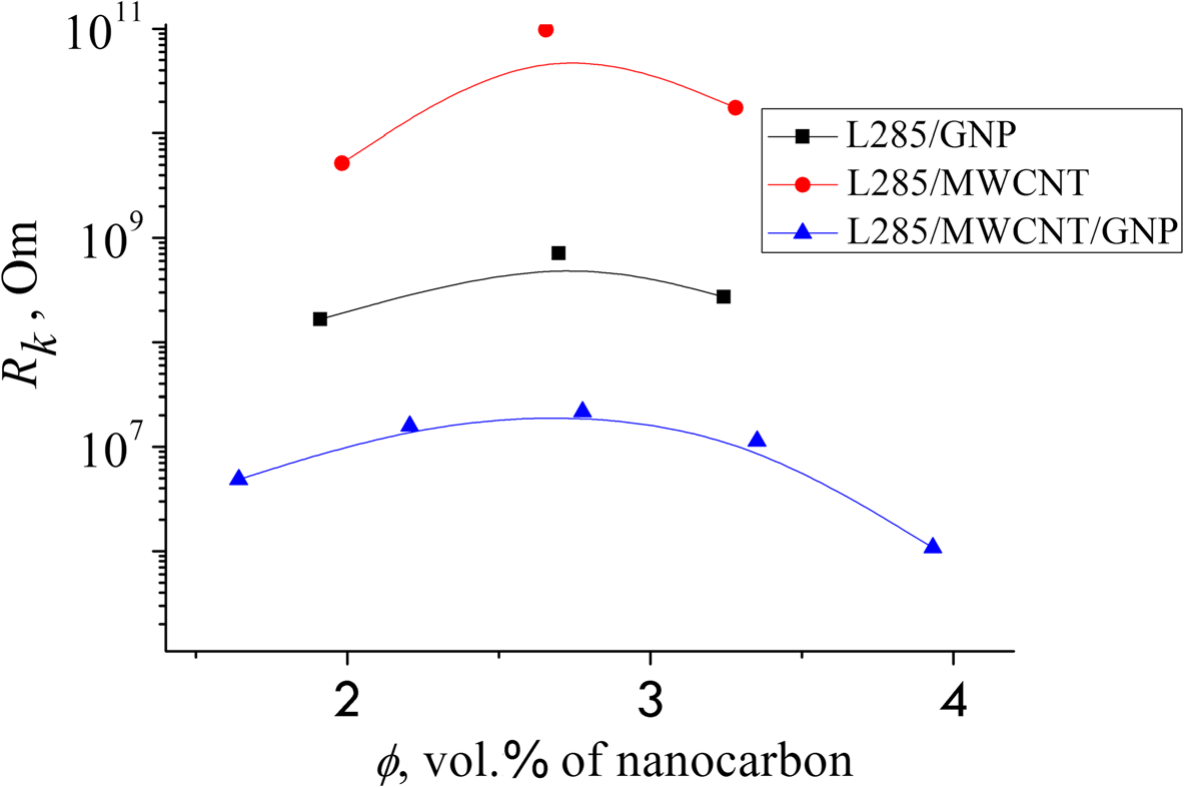
Contact resistance *R*_*k*_ of the studied CMs, which was calculated by using Eq. (3)

In terms of the proposed model, followed values were calculated: concentration dependence of the electrical conductivity *σ*_dc_ (*ϕ*), contact electric resistance *R*_*к*_, and number of uninterrupted chains *N* ^*^_chain_in_CM_ (see Fig. 6, Table 3).

**Table 3:** Parameters of the polymer/nanocarbon CM with different concentration of the conductive filler

CM	*ϕ*, vol%	*N* ^*^_chain_in_CM_, cm^−3^	*R*_*к*_ (293K), Ω
L285/GNP	1.90	6.17 × 10^+3^	1.66 × 10^+8^
	2.70	9.90 × 10^+5^	7.10 × 10^+8^
	3.20	3.12 × 10^+6^	2.71 × 10^+8^
L285/MWCNT	2.00	5.72 × 10^+6^	5.19 × 10^+9^
	2.70	2.82 × 10^+8^	9.73 × 10^+10^
	3.30	1.13 × 10^+9^	1.75 × 10^+10^
L285/MWCNT/GNP	1.60	3.55 × 10^+3^	4.88 × 10^+6^
	2.20	1.10 × 10^+5^	1.58 × 10^+7^
	2.70	5.02 × 10^+5^	2.18 × 10^+7^
	3.40	1.35 × 10^+6^	1.14 × 10^+7^
	4.00	1.26 × 10^+6^	1.08 × 10^+6^

As we can see from the Table 3, the value of the contact electric resistance *R*_*к*_ exhibits minimum at 10^+6^–10^+7^ Ω for hybrid filler and maximum near 10^+9^–10^+10^ Ω for the CM based on CNT. The CNT-based CMs have 300 (600) times bigger total amount of uninterrupted chains *N* ^*^_chain_in_CM_ than the CMs based on GNP (hybrid filler) with concentration 2.70 vol%. Electrical conductivity of L285/MWCNT/GNP is higher than the electrical conductivity of a binary CM (Fig. 2).

On the other hand, formation of the conducting network of nanocarbon particles would not necessary cause high electrical conductivity. According to [31], numerical simulations showed that contact resistance between different nanotubes varies from 100 kOhm to 3.4 MOhm and strongly depends on the atomic structure of the contact surface and structural relaxation of the particles. Contact resistance may appear during the formation of the dielectric layer between contact points of the filler components (due to the wetting). This dielectric layer causes degrade of the conductivity and stimulates tunneling of the charge carriers [32].

Electric resistance *R*_*к*_ between two contacting particles can be evaluated as follows [33]:

4 $$ {R}_{k\left(\mathrm{tunel}\right)}=\frac{V}{w\cdot j}=\frac{h^2\delta}{w{ e}^2\sqrt{2 m\lambda}} \exp \left(\frac{4\pi \delta}{h}\sqrt{2 m\lambda}\right) $$

where *δ* is the thickness of the polymer layer; *j* is the density of the tunnel current; *V* is the potential difference; *e* and *m* are the charge and mass of an electron, respectively; *h* is the Planck constant; *λ* is the height of the barrier [34–36] (in case of the epoxy *λ* ≈ 1 eV [33]); and *w* is the cross-sectional tunneling value.

**Fig. 7:**
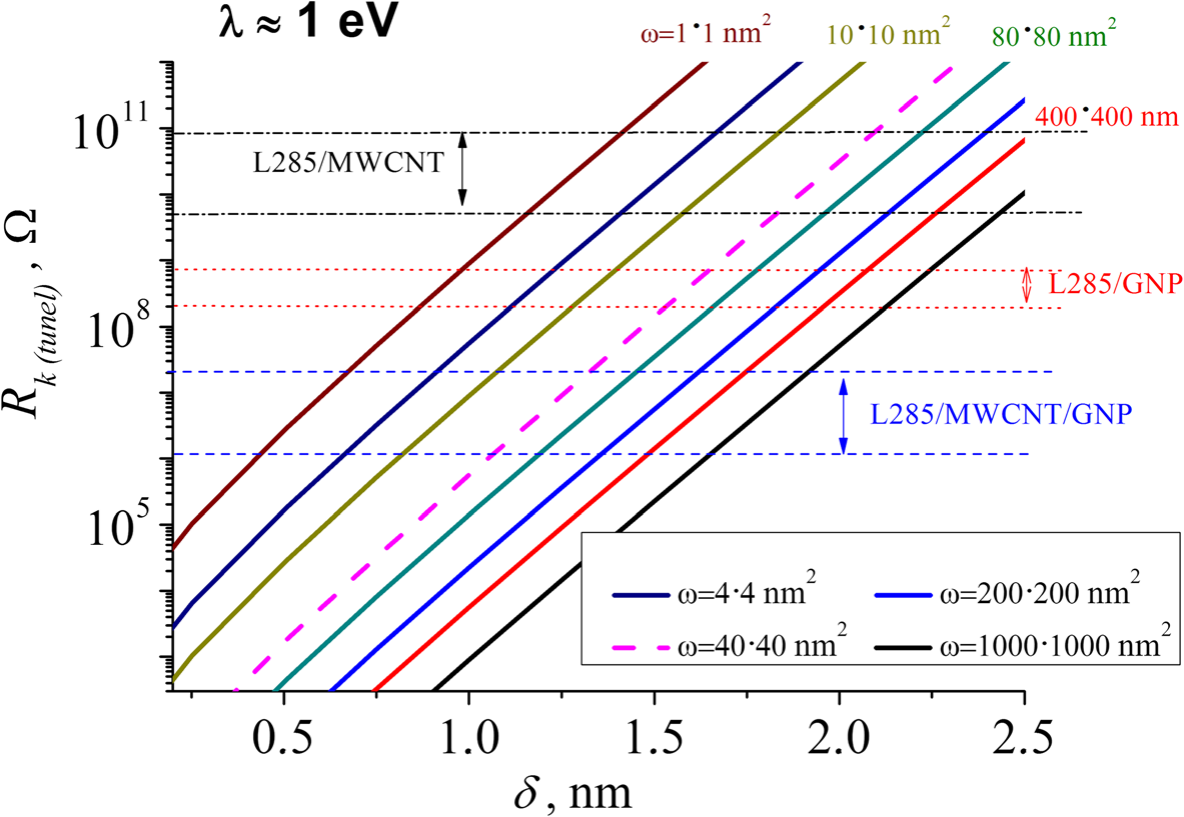
The dependences of the contact resistance on the thickness of the polymer layer between the filler particles for various values of the cross-sectional tunneling *w*

Figure 7 shows that the values of the electric contact resistance in case of the tunneling mechanism of the conductivity depend on the distance (thickness of polymer layer) between the filler particles for a variety of cross-sectional tunneling values (calculated using the expression 4).

Figure 7 demonstrates the strong dependence of the *R*_*k*(tunel)_ on *δ*. Thus, with increasing *δ* values from 0.5 to 2.5 nm, *R*_*k*(tunel)_ value grew by nine orders of magnitude regardless of the tunnel cross section *w*.

Numerical simulations showed that the distance between GNP particles slowly degrades from 1.63 to 1.53 nm for the *w* = 40 × 40 nm^2^, which witnesses about the absence of the tunneling and destruction of the conductive chain for *δ* ≤ 1.63 nm.

In the case of CNT-based CMs, decreasing of the *δ* from 1.83 to 1.57 nm with *w* = 10 × 10 nm^2^ is caused by the smaller sizes of the CNT compared to the GNP sizes. We assume that *δ* of GNP-based CM is smaller due to the ultraviolet treatment (cleaning of the surface from the particle functional groups) and better contact between the polymer and filler particles [22, 23].

CMs with a hybrid filler demonstrate decreasing of *δ* from 1.07 to 0.82 nm (from 1.32 to 1.05 nm) for *w* = 10 × 10 nm^2^ (*w* = 40 × 40 nm^2^).

As we can see from Fig. 7 and Table 3, the polymer layer thickness *δ* for CMs with a hybrid filler is the smallest regardless of the magnitude of the tunneling cross section, despite higher amount of a conductive chains in CNT-based CMs.

## Conclusions

It has been found that two percolation thresholds are formed in polymer solution with nanocarbon with low viscosity. The first is a quasi-dynamic percolation transition which nature is associated with the movement of light separate nanocarbon particles until the mixture is cured. The second percolation transition is static, described by the classical theory of percolation and allowed us to calculate the number of conductive chains and the contact resistance between the filler particles in terms of the model of effective electrical resistivity. It has been found that there is a synergistic effect above the percolation threshold for CMs with a hybrid filler (namely, with the carbon nanotubes and graphite nanoplatelets). It has been shown that a synergistic effect for the CMs with a hybrid filler is possible due to reducing contact resistance between the particles of both fillers, which may be associated with a decrease of the polymer layer thickness between the particles and appearance of moderate amount of the conductive chains with increase of the number of particles involved in a single chain.
